# The Effect of Induced Optimism on Situational Pain Catastrophizing

**DOI:** 10.3389/fpsyg.2022.900290

**Published:** 2022-06-23

**Authors:** Johanna Basten-Günther, Madelon L. Peters, Stefan Lautenbacher

**Affiliations:** ^1^Department of Physiological Psychology, University of Bamberg, Bamberg, Germany; ^2^Department of Clinical Psychological Science, Maastricht University, Maastricht, Netherlands

**Keywords:** pain, pain catastrophizing, optimism, resilience, positive psychology

## Abstract

**Background:**

There is broad evidence that optimism is associated with less pain, while pain catastrophizing leads to increased pain. The aim of this study was to examine whether experimentally induced optimism can reduce situational pain catastrophizing and whether this relation is moderated by dispositional optimism and/or dispositional pain catastrophizing.

**Methods:**

Situational pain catastrophizing during two thermal stimulations was measured in 40 healthy participants with the *Situational Catastrophizing Questionnaire (SCQ)*. Between the two stimulations, the *Best Possible Self (BPS)* imagery and writing task was performed to induce situational optimism in the experimental group while the control group wrote about their typical day. Questionnaires were administered to assess dispositional optimism [*Life Orientation Test-Revised (LOT-R)*] and dispositional pain catastrophizing [*Pain Catastrophizing Scale (PCS)*].

**Results:**

There was a significant interaction between the optimism induction and trait pain catastrophizing: the association of trait pain catastrophizing with state pain catastrophizing was weakened after the optimism induction. No overall effect of induced optimism on situational pain catastrophizing and no significant moderating influence of trait optimism were found.

**Conclusion:**

The state optimism induction apparently counteracted the manifestation of dispositional pain catastrophizing as situational pain catastrophizing. This implies that high trait pain catastrophizers may have especially benefitted from the optimism induction, which is in line with resilience models stressing the buffering role of optimism.

## Introduction

Optimism—defined as positive expectancies concerning the future (Scheier and Carver, 1985)—is known to have pain-dampening effects in experimental as well as in acute and chronic clinical pain (for overviews, see Garofalo, 2000; Goodin and Bulls, 2013; Basten-Günther et al., 2019). Pain catastrophizing is a negative mental set during actual or anticipated pain, consisting of rumination, magnification and feelings of helplessness when in pain (Sullivan, 2009; Campbell et al., 2010a; Pulvers and Hood, 2013). Pain catastrophizing leads to higher pain reports (Adams et al., 2007; Sullivan, 2009; Campbell et al., 2010b). Both optimism and pain catastrophizing are conceptualized as having a trait component (dispositional optimism/pain catastrophizing) and a state component (situational optimism/pain catastrophizing; Kluemper et al., 2009; Quartana et al., 2009; Campbell et al., 2010a).

As pain catastrophizing and optimism influence pain experience in opposite ways, it is interesting to examine how these two variables interact. It has been assumed that optimists are less likely to engage in pain catastrophizing, which in turn leads to lower pain reports, i.e., that the negative association between optimism and pain is fully or partially mediated by pain catastrophizing (Hood et al., 2012; Goodin et al., 2013; Hanssen et al., 2013; Pulvers and Hood, 2013, for an overview). The present study therefore aims at exploring whether an experimental induction of state optimism can successfully reduce the situational pain catastrophizing occurring during painful heat stimulation. Furthermore, we examine whether the levels of dispositional optimism and dispositional pain catastrophizing moderate this relationship. In accordance with theories on resilience factors (for example, Catalano et al., 2011), situational optimism might act as a “buffer” by preventing or attenuating the manifestation of trait pain catastrophizing as situational pain catastrophizing in a given pain situation. This implies that high dispositional pain catastrophizers might benefit more from the optimism induction as regards their situational pain catastrophizing than low dispositional pain catastrophizers, who would already show low situational catastrophizing responses without the optimism manipulation.

As regards dispositional optimism, there have so far not been found any differences in the responsiveness to an optimism induction between participants with high vs. low trait optimism (Harrist et al., 2007; Peters et al., 2010; Hanssen et al., 2013). It is thinkable that analogously to low dispositional pain catastrophizers, high trait optimists might benefit less from an induction of additional optimism as they are already sufficiently optimistic and in consequence less prone to pain catastrophizing even without an additional optimism “boost.” In this sense, high trait optimists would show weaker changes in situational pain catastrophizing after the experimental induction of state optimism.

Firstly, we hypothesize that experimentally induced situational optimism leads to reduced situational pain catastrophizing. Secondly, we hypothesize that the influence of the optimism induction is stronger in high dispositional pain catastrophizers. Thirdly, we hypothesize that the influence of the optimism induction is weaker in individuals with high dispositional optimism.

## Materials and Methods

### Participants

A total of 40 healthy, pain-free individuals [20 men and 20 women, 10 from each decade between 20 and 60; mean (±SD) age 39.9 ± 13.5 years] participated in the current study. The participants were recruited *via* advertisements in the local newspaper (Bamberg, Germany). To ensure normal affectivity, current psychological disorders as assessed *via* self-report were an exclusion criterion. Participants were asked not to take alcohol or analgesic and psychotropic drugs on the day of the experiment. All participants provided informed consent and received monetary compensation. The study protocol was approved by the ethics committee of the University of Bamberg (Bamberg, Germany).

### Procedure

#### General Protocol

We chose an experimental design comparing situational pain catastrophizing in an experimental group receiving an optimism manipulation with a control group receiving a neutral state induction. Part of the data have been published before in an analysis of different outcome variables, namely self-report pain ratings and facial expression of pain (Basten-Günther et al., 2021). Group assignment was randomized. The two groups were balanced with regard to age and sex and—for female participants not using hormonal contraceptives—with regard to phase of menstrual cycle (follicular, ovulatory and luteal) in order to control for mood swings over the course of the menstrual cycle (Natale and Albertazzi, 2006). Situational pain catastrophizing was measured with reference to two identical painful stimulations applied before (pre measurement) and after the optimism/control manipulation (post measurement).

**Figure 1:**
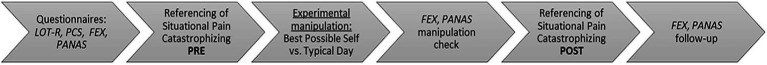
General protocol of the experiment. LOT-R, Life Orientation Test-Revised Version; PCS, Pain Catastrophizing Scale; FEX, Future Expectancies Scale; PANAS, Positive and Negative Affect Schedule; and SCQ, Situational Catastrophizing Questionnaire.

The experiment consisted of one session taking place at 1.30 pm for one half of the participants and at 3.30 pm for the other half. Participants from the experimental and the control group were distributed equally across the two points of time in order to control for fluctuations of mood across the day (Murray et al., 2002). After providing informed consent and filling out questionnaires to assess trait optimism, trait pain catastrophizing and baseline state optimism (see below), participants underwent two identical blocks of painful heat stimulation which served as a standardized reference of situational pain catastrophizing. After each block, participants rated the catastrophizing thoughts they had experienced during the painful stimuli which they had just received, on the Situational Catastrophizing Questionnaire (SCQ; see below). Results of pain variables (ratings of pain intensity and pain unpleasantness as well as facial expression of pain) recorded during this stimulation have recently been reported by Basten-Günther et al. (2021). In between the two pain blocks, which will be described in detail below, the experimental manipulation (optimism-inducing vs. neutral writing task) was executed, which acted as independent variable. The state questionnaires Positive and Negative Affect Schedule (PANAS) and Future Expectancies Scale (FEX), which served as a manipulation check, were filled out three times in order to record induced changes in affect and situational optimism: before the first pain block (baseline), immediately after the optimism intervention and after the second pain block (follow-up). After roughly 2 h, the participants were thanked and debriefed and the session was concluded (Figure 1).

#### Measurement of Situational Pain Catastrophizing

To reference situational pain catastrophizing to a standardized pain situation, participants underwent two blocks of heat stimulation (one before and one after the experimental manipulation of optimism), which each consisted of 10 painful and 10 non-painful phasic heat stimuli in the same random order. Each stimulus had a duration of 5 s. Using an experimental procedure to trigger acute situational pain catastrophizing—rather than asking participants to reference a past everyday pain experience—prevents memory effects and variation in referenced pain events. Most importantly, it allows for a standardized pain stimulation which can be subjected to a pre–post-manipulation comparison.

The stimuli were applied to the left volar forearm with a 30 × 30 mm contact thermode. To ensure that temperature intensities were perceived as painful but not too painful in all participants, temperature intensities were tailored to the individual pain threshold, which was determined by the method of adjustment (for example, Horn et al., 2012) in four trials. Both painful (+3°C above the pain threshold) and non-painful (−1°C below the pain threshold) intensities were applied in a random order to sustain participants’ vigilance and to prevent changes in pain sensitization which might in turn alter situational pain catastrophizing and in the following distort or conceal the optimism effects on situational pain catastrophizing.

#### Optimism Manipulation

In between the two blocks of heat stimulation, the experimental optimism induction was performed. Optimism was induced by the *Best Possible Self* task (BPS), a positive future thinking technique based on work by King (2001). BPS has been proven effective in increasing optimism temporarily (Peters et al., 2010, 2016; Hanssen et al., 2013). Participants were instructed to carry out a writing and imagery exercise. Half of the participants were assigned to the BPS condition (*n* = 20), which required them to write about their life in the future where everything had turned out for the best. The other half of the participants were assigned to the control condition (*n* = 20), whose task consisted in writing about a typical day (TD). The instructions for BPS and TD were as follows (cf. Sheldon and Lyubomirsky, 2006).

- BPS condition:
*Thinking about your best possible self means that you imagine yourself in the future, after everything has gone as well as it possibly could. You have worked hard and succeeded at accomplishing all the goals of your life. Think of this as the realization of your dreams, and that you have reached your full potential.*
- TD condition:
*Thinking about your typical day means that you take notice of ordinary details of your day that you usually do not think of. These might include particular classes or meetings you attend to, people you meet, things you do, typical thoughts you have during the day. Think of this as moving through your typical day, hour after hour.*

Both manipulations had the same procedural format: participants were requested to think for 1 min about what to write, then to write uninterrupted for 15 min, followed by 5 min of imagining the story they had just been writing. Instructions were given both verbally and in writing. The manipulation check followed immediately by asking the participants to complete the FEX and PANAS a second time and to answer three questions about the quality and valence of their writing and imaginations (*Quality of imagery*, Peters et al., 2010).

#### Questionnaires

##### Situational Catastrophizing Questionnaire

Situational pain catastrophizing with reference to the two painful stimulations was assessed with the German version of the SCQ (Edwards et al., 2006) which was translated by the authors and has been applied before in several studies (for example, Horn-Hofmann et al., 2018; Karmann et al., 2018; Stroemel-Scheder et al., 2019). There is evidence that situational catastrophizing as measured by the SCQ correlates significantly stronger with pain reports than dispositional pain catastrophizing as measured by the Pain Catastrophizing Scale (PCS; Sullivan et al., 1995; Dixon et al., 2004; Edwards et al., 2005; Campbell et al., 2010a). The SCQ is an adaptation of the PCS and consists of six items referring to catastrophizing thoughts and feelings during a specific noxious stimulation which was applied before filling out the questionnaire. The items tap the three dimensions of pain catastrophizing (rumination, for example: “I could not stop thinking about how much it hurt.”; magnification: “I felt that the procedures were awful.”; helplessness: “I felt that I could not stand it.”) and are rated on a five-point scale, with the end points “not at all” and “all the time.” Cronbach’s alpha in this study was *α* = 0.91 for the measurement after the first block of stimulation and *α* = 0.94 for the measurement after the second block of stimulation.

##### Pain Catastrophizing Scale

A German translation (Meyer et al., 2008) of the PCS (Sullivan et al., 1995) was used to assess catastrophic thinking related to pain. Participants are instructed to reflect on thoughts or feelings during past painful experiences. The scale comprises 13 items on the subscales rumination, magnification and helplessness, which are rated on a five-point scale, with the end points “not at all” and “all the time.” The PCS has been widely used in research on pain catastrophizing and has been shown to have high internal consistency. Internal consistency in the present case was Cronbach’s *α* = 0.91.

##### Life Orientation Test Revised

The validated German version (Krohne et al., 1996) of the Life Orientation Test-Revised (LOT-R; Scheier et al., 1994) was used to assess the level of dispositional optimism. The LOT-R has 10 items which are rated on a five-point Likert scale, ranging from 0 (“strongly disagree”) to 4 (“strongly agree”). There are three positively phrased items (optimism subscale), three negatively phrased items (pessimism subscale), and four filler items. A total trait optimism score is calculated over the six items with either positive or negative content after reversing the negatively phrased items. Internal consistency as measured by Cronbach’s alpha was *α* = 0.76.

##### Future Expectancies Scale

The FEX (Hanssen et al., 2013) was administered to assess state optimism. A German version of the questionnaire was used, which was translated in a standard “forward-backward” procedure and applied in a prior study by the authors (Peters et al., 2016). The FEX consists of 10 statements describing a positive future event and 10 statements describing a negative future event. Participants rated the likelihood that they will experience each specific event on a seven-point Likert scale, ranging from 1 (“not at all likely to occur”) to 7 (“extremely likely to occur”). The FEX has previously been demonstrated to be responsive to optimism manipulations (Hanssen et al., 2013; Boselie et al., 2014). The subscores FEX positive and FEX negative were used for further analyses. Internal consistency at the three assessment times ranged from Cronbach’s *α* = 0.89 to *α* = 0.91 for the subscale FEX positive and from *α* = 0.83 to *α* = 0.87 for the subscale FEX negative.

##### Positive and Negative Affect Schedule

Mood was assessed with the PANAS (Watson et al., 1988). The PANAS consists of 20 items measuring positive (10 items) and negative (10 items) affect. Participants indicate the degree to which a certain feeling is present at that moment on a five-point Likert scale ranging from 1 (“not at all”) to 5 (“extremely”). The subscores PANAS positive (PANAS_PA) and PANAS negative (PANAS_NA) were used for further analyses. For the PANAS, a validated German version (Glaesmer et al., 2008) was used. Internal consistency at the three assessment times ranged from Cronbach’s *α* = 0.86 to *α* = 0.93 for PANAS_PA and from *α* = 0.66 to *α* = 0.83 for PANAS_NA.

##### Quality of Imagery

Two visual analogue scales (0–100 mm; Peters et al., 2010) were used to rule out qualitative (in contrasts to content-related) differences in participants’ imagery between the BPS and the TD group (Hanssen et al., 2013): “How well could you imagine yourself in the situation you described in your writing” and “How vivid were the pictures you imagined?.” A third VAS (“How negative or positive were your imaginations?”) was administered to rule out that imaginations and writing content were equally positive in the TD group as in the BPS group.

### Statistical Analyses

In order to examine the effect of the optimism induction on situational pain catastrophizing, we tested for a time x condition interaction effect in a 2 × 2 repeated measures ANOVA. Experimental condition was entered as fixed factor.

To test moderating effects of dispositional pain catastrophizing and dispositional optimism, preliminary bivariate correlations were computed to test for associations between the SCQ score and the LOT-R and PCS score, respectively. In case of significant correlations, separate analysis of covariances (ANCOVAs) were performed with *LOT-R* or *PCS* as covariates. Second-order interaction effects (time x condition x *LOT-R*/*PCS*) were specified in the model. In case of significant second-order interactions, within group regressions of SCQ change scores on LOT-R/PCS were performed to determine the direction on the interaction.

In order to assess differences in demographic or baseline variables between the BPS and the TD group, independent samples *t*-tests comparing the two groups were applied. To control whether the optimism induction was successful, a 3 × 2 (time x group) repeated measures ANOVA was computed for each FEX/PANAS subscale. Independent samples *t*-tests were used to investigate group differences as regards the quality of writing and visualization (Basten-Günther et al., 2021). All analyses were conducted with SPSS 24 and the alpha-level was 0.05 throughout.

## Results

### Descriptive Statistics

Means and SDs for demographic variables, dispositional optimism (*LOT-R*), dispositional pain catastrophizing (*PCS*) and situational pain catastrophizing (*SCQ*) are shown in Table 1. The LOT-R mean score is exactly the same as the population-based norm recently reported by Schou-Bredal et al. (2017). The *PCS* score is similar to the ones found in prior studies (for example, Kunz et al., 2016; Horn-Hofmann et al., 2017; Basten-Günther et al., 2021).

**Table 1:** Demographics and trait measures of optimism and pain catastrophizing and situational pain catastrophizing.

Sex male	TD		BPS		*t*-test for independent samples (TD vs. BPS)	
	*n* = 20		*n* = 20			
	10 (50%)		10 (50%)			
	Mean	*SD*	Mean	*SD*	*t*	*p*
Age *(years)*	40.50	11.69	40.20	12.60	0.08	0.94
*LOT-R*	16.40	3.17	18.05	4.06	1.43	0.16
*PCS*	15.50	7.94	11.83	8.04	1.45	0.15
*SCQ pre*	6.30	5.74	7.40	6.06	0.59	0.56
*SCQ post*	7.10	7.01	8.55	6.40	0.68	0.50

BPS, Best Possible Self (experimental group); TD, Typical Day (control group); LOT-R, Life Orientation Test-Revised Version; PCS, Pain Catastrophizing Scale; and SCQ, Situational Catastrophizing Questionnaire.

### Randomization Check

The optimism and control group did neither significantly differ in their pain threshold nor in any demographic variable or baseline state measurement (first assessment of *SCQ*, *FEX*, and *PANAS*). For this reason, neither of these variables was controlled for in subsequent AN(C)OVAs (Basten-Günther et al., 2021).

### Manipulation Check

As already reported in Basten-Günther et al. (2021), there was a significant time x group interaction effect for PANAS_PA [subscale positive affect; *F*(2,76) = 3.89, *p* = 0.03 *η_p_*^2^ = 0.09] and FEX_pos [subscale positive future expectancies; *F*(2,76) = 3.07, *p* = 0.05, *η_p_*^2^ = 0.08].

Within-condition analyses of repeated measurements with planned contrasts indicated that in the BPS condition, FEX_pos was significantly larger at the post-manipulation assessment [*F*(1,19) = 6.5, *p* = 0.02, *η_p_*^2^ = 0.255] compared to the pre-manipulation assessment. In the TD group, there were no differences between the three assessments. Corresponding analyses for the PANAS_PA scale showed a significant decrease of positive affect in the TD group at the post-manipulation assessment compared to baseline [*F*(1,19) = 7.6, *p* = 0.01, *η_p_*^2^ = 0.287] whereas positive affect in the BPS group remained stable after the manipulation [*F*(1,19) = 1.2, *p* = 0.29, *η_p_*^2^ = 0.058]. There were no significant differences between the three assessment times in the negative subscales of FEX and PANAS.

The differences between the groups after the experimental manipulation suggests that the optimism induction was successful. This is in accordance with prior studies using the same paradigm (Peters et al., 2010, 2016; Hanssen et al., 2013).

The groups did not significantly differ in the VAS about the quality (BPS: *M* = 77.35, *SD* = 15.09, TD: *M* = 74.05, SD = 21.75; *t* = 0.56; *p* = 0.58) and the vividness (BPS: *M* = 77.15, *SD* = 22.73, TD: *M* = 69.25, *SD* = 22.09; *t* = 1.12; *p* = 0.27) of their imaginations. This is in accordance with prior research (Hanssen et al., 2013). On the third question asking the emotional valence, the BPS group scored, as expected, significantly higher than the TD group (BPS: *M* = 86.60, *SD* = 14.73, TD: *M* = 61.15, *SD* = 25.09; *t* = 3.91, *p* < 0.005; Basten-Günther et al., 2021).

### Overall Influence of Optimism Induction on Situational Pain Catastrophizing

**Figure 2:**
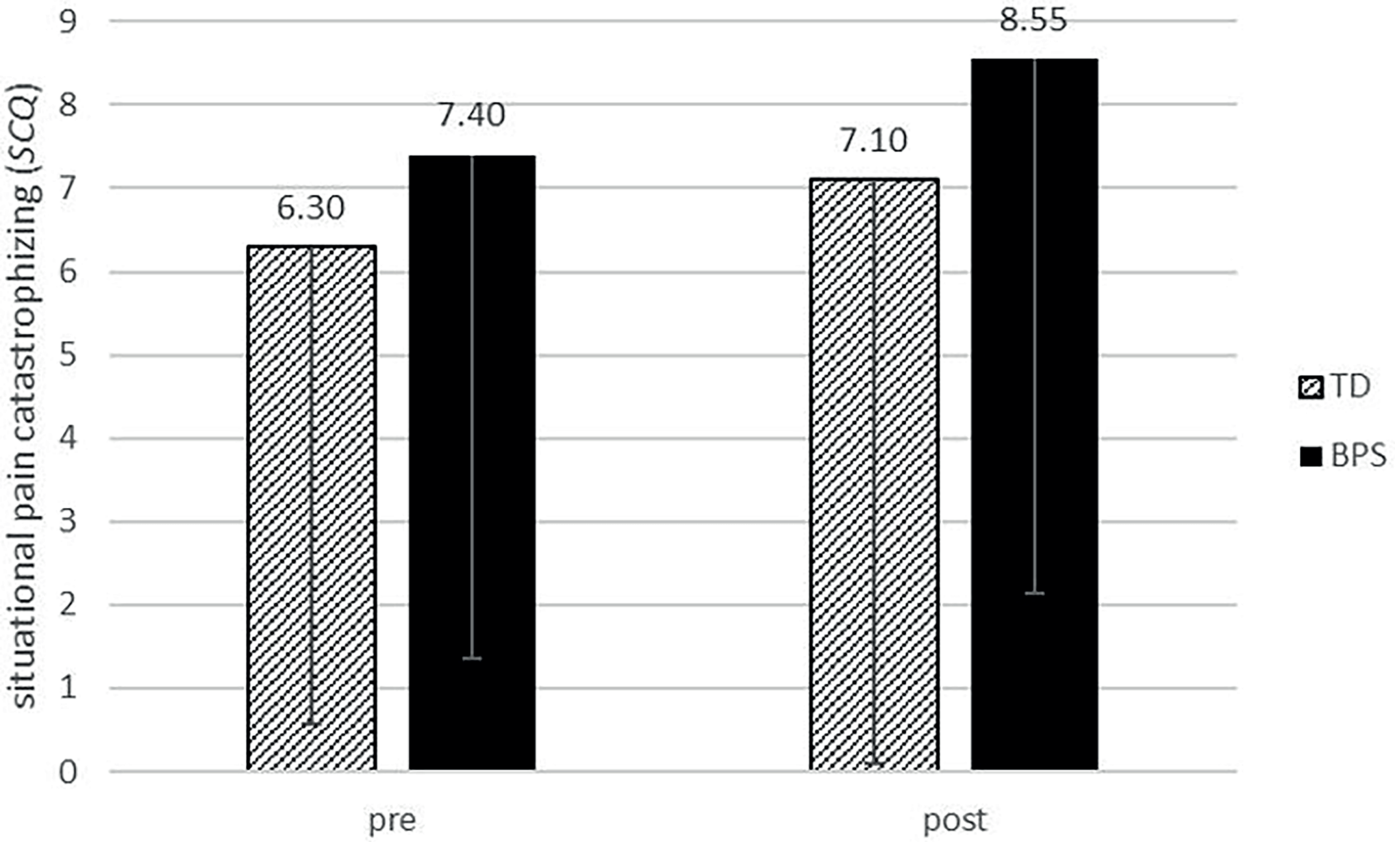
Mean score of overall situational pain catastrophizing (SCQ) depending on experimental condition and time of measurement. Error bars = −1 SD. TD, typical day and BPS, Best Possible Self.

As regards the overall influence of the optimism induction on situational pain catastrophizing independent of trait levels, the 2 × 2 repeated measures ANOVA did not show a significant time x condition interaction effect [*F*(1,38) = 0.101, *p* = 0.752, *η_p_*^2^ = 0.003]. This means that there was no overall effect of induced optimism on situational pain catastrophizing across all participants. Figure 2 shows the level of situational pain catastrophizing depending on time of measurement and experimental condition. There was a non-significant overall increase in the SCQ from the pre- to the post-measurement across both groups (*M* = 0.975, *t* = 1.794, *p* = 0.081).

### Influence of Optimism Induction on Situational Pain Catastrophizing Dependent on Level of Dispositional Pain Catastrophizing

Preliminary analyses revealed significant correlations between PCS scores and SCQ pre- and post-scores (whole sample: pre: *r* = 0.475, *p* = 0.002; post: *r* = 0.365, *p* = 0.021; TD group: pre: *r* = 0.490, *p* = 0.028; post: *r* = 0.529, *p* = 0.016; BPS group: pre: *r* = 0.533, *p* = 0.016; post: *r* = 0.269, *p* = 0.252). These linear relations are also shown in regression graphs of SCQ on PCS in supplement A. Upon visual examination of p–p-plots, residuals were approximately normally distributed, with the exception of the BPS post-measurement, which seems to reflect the disparition of the association between PCR and SCQ after the optimism induction.

Subsequently, 2 × 2 repeated measures ANCOVA was conducted to examine the influence of the optimism induction on situational pain catastrophizing dependent on participants’ level of trait pain catastrophizing. The ANCOVA revealed a significant main effect of dispositional pain catastrophizing [*PCS*; *F*(1,36) = 10.399, *p* = 0.003, *η_p_*^2^ = 0.224]—higher *PCS* scores were associated with higher *SCQ* scores—, a close to significant trend of the time x condition interaction [*F*(1,36) = 4.027, *p* = 0.052, *η_p_*^2^ = 0.101] and a significant time x condition x *PCS* second-order interaction effect [*F*(1,36) = 4.902, *p* = 0.033, *η_p_*^2^ = 0.120; Table 2].

**Table 2:** *F*-Tests, value of *p* and effect sizes of the analysis of covariance (ANCOVA) with the factors condition (between-subjects, levels: BPS and TD), time (within-subject, levels: pre and post) and the covariate dispositional pain catastrophizing (Pain Catastrophizing Scale: *PCS*) for the dependent variable situational pain catastrophizing.

	Condition	Time	*PCS*	Condition x *PCS*	Time x *PCS*	Time x condition	Time x condition x *PCS*
Situational Pain Catastrophizing (*SCQ*)	*F*(1,36) = 1.286; *p* = 0.264; *η_p_*^2^ = 0.034	*F*(1,36) = 0.1.270; *p* = 0.267; *η_p_*^2^ = 0.034	*F*(1,36) = 10.399; ***p* = 0.003**; ***η***_***p***_^**2**^ **= 0.224**	*F*(1,36) = 0.215; *p* = 0.646; *η_p_*^2^ = 0.006	*F*(1,36) = 0.305; *p* = 0.584; *η_p_*^2^ = 0.008	*F*(1,36) = 4.027; *p* = 0.052; *η_p_*^2^ = 0.101	*F*(1,36) = 4.902; ***p* = 0.033**; ***η***_***p***_^**2**^ **= 0.120**

Significant effects (*p* ≤ 0.05) are marked by bold text and gray-shaded background.

**Figure 3:**
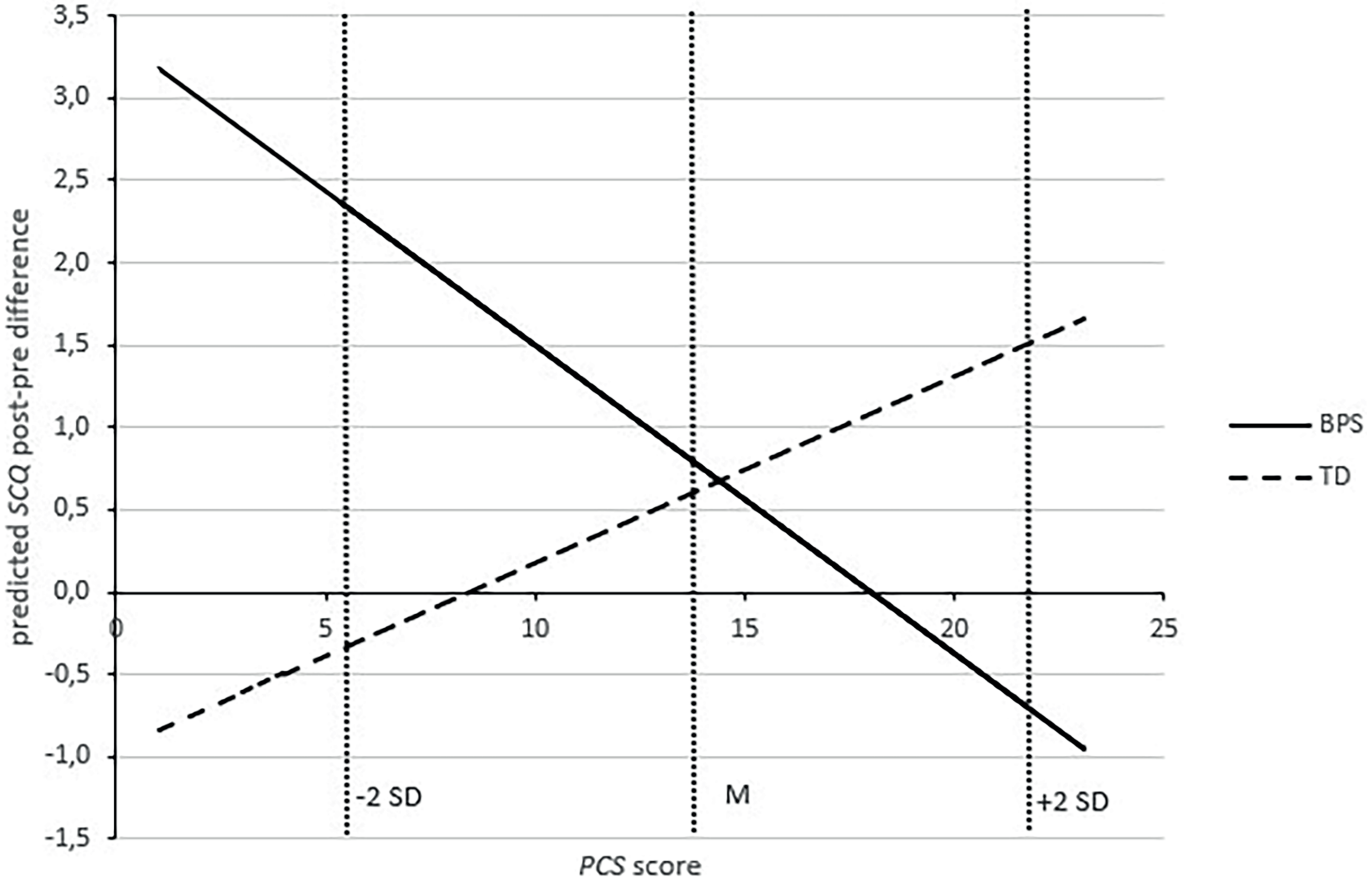
Regression of change in situational pain catastrophizing (post minus pre difference) on dispositional pain catastrophizing (PCS score). Estimated SCQ values at means (M) and plus/minus two SDs of PCS score are indicated by vertical lines. Positive values indicate an increase in SCQ from the pre to the post measurement, while negative values indicate a decrease. BPS, Best Possible Self (experimental group). TD, Typical Day (control group). SCQ, Situational Catastrophizing Questionnaire. PCS, Pain Catastrophizing Scale.

Figure 3 illustrates this second-order interaction by showing pre-post SCQ change scores regressed on the PCS score for each group. As can be seen in Figure 3, high PCS scores predicted an increase from the pre- to the post-measurement in the BPS group, but a difference of around zero in the TD group. The SCQ level of low pain catastrophizers (low values of PCS) remains approximately constant in the TD condition, while an increase from pre to post is predicted in the BPS task.

Corresponding to the main effect of PCS, low dispositional pain catastrophizers scored several points lower in the SCQ than high dispositional pain catastrophizers across both groups and both times of measurement.

To sum it up, a significant time x condition x *PCS* second-order interaction effect was found. Descriptively, there was a less pronounced pre–post-SCQ increase in high trait pain catastrophizers of the optimism group compared to high trait pain catastrophizers of the control group.

### Influence of Optimism Induction on Situational Pain Catastrophizing Dependent on Level of Dispositional Optimism

Preliminary analyses indicated that the LOT-R score was significantly correlated with the SCQ pre score (*r* = −0.342, *p* = 0.031), but not the SCQ post score (*r* = −0.178, *p* = 0.273; TD group: pre: *r* = −0.224, *p* = 0.343; post: *r* = −0.161, *p* = 0.497; BPS group: pre: *r* = −0.412, *p* = 0.071; post: *r* = −0.159, *p* = 0.504). Due to the former, a 2 × 2 repeated measures ANCOVA was conducted to examine the influence of the optimism induction on situational pain catastrophizing dependent on participants’ level of trait optimism. As shown in Table 3, there was neither a significant main effect of the LOT-R [*F*(1,36) = 2.144, *p* = 0.152, *η_p_*^2^ = 0.056] nor a significant time x condition x LOT-R second order interaction effect [*F*(1,36) = 0.102, *p* = 0.320, *η_p_*^2^ = 0.027]. Thus, dispositional optimism did not have a direct association with situational pain catastrophizing and did not alter the effect of the optimism induction on situational pain catastrophizing.

**Table 3:** *F*-Tests, value of *p* and effect sizes of the ANCOVA with the factors condition (between-subjects, levels: BPS and TD), time (within-subject, levels: pre and post) and the covariate dispositional optimism (Life Orientation Test-Revised: *LOT-R*) for the dependent variable situational pain catastrophizing.

	Condition	Time	*LOT-R*	Condition x *LOT-R*	Time x *LOT-R*	Time x condition	Time x condition x *LOT-R*
Situational Pain Catastrophizing (*SCQ*)	*F*(1,36) = 0.023; *p* = 0.880; *η_p_*^2^ = 0.001	*F*(1,36) = 0.771; *p* = 0.386; *η_p_*^2^ = 0.021	*F*(1,36) = 2.144; *p* = 0.152; *η_p_*^2^ = 0.056	*F*(1,36) = 0.009; *p* = 0.926; *η_p_*^2^ < 0.001	*F*(1,36) = 1.734; *p* = 0.196; *η_p_*^2^ = 0.046	*F*(1,36) = 0.728; *p* = 0.399; *η_p_*^2^ = 0.020	*F*(1,36) = 1.018; *p* = 0.320; *η_p_*^2^ = 0.027

## Discussion

The aim of the present study was to examine the effect of an experimental optimism induction on situational pain catastrophizing (SCQ; Hypothesis 1) and to explore the moderating roles of dispositional pain catastrophizing (PCS; Hypothesis 2) and dispositional optimism (LOT-R; Hypothesis 3). There was no direct effect (Hyp.1) of the optimism induction and no moderating influence of dispositional optimism (Hyp.3) on situational pain catastrophizing. As regards the moderating role of dispositional pain catastrophizing, there was a significant time x condition x PCS interaction effect, suggesting a stronger influence of the optimism induction on situational pain catastrophizing in high pain catastrophizers This finding supports Hypothesis 2. The discussion will follow the order of hypotheses.

### Direct Influence of Optimism Induction on Situational Pain Catastrophizing (Hyp.1)

Contrary to our hypothesis, the experimental optimism induction did not impact on situational pain catastrophizing. Thus, our results do not support prior studies which found a negative association between optimism and pain catastrophizing. Two of these studies (Hood et al., 2012; Goodin et al., 2013) only applied trait questionnaires without an experimental manipulation of optimism or a situational measure of pain catastrophizing and may therefore be less comparable. The other one (Hanssen et al., 2013) used the BPS task and a measure of state pain catastrophizing. In this study, pain catastrophizing during a 2-min cold pressor task was measured. As has been argued before (Basten-Günther et al., 2021), the longer duration of this pain may have provided more opportunity for optimistic attitudes to unfold their effect *via* cognitive appraisal processes than the 5 s stimuli in our study. Catastrophizing thoughts may thus have been prevented more effectively. The pain ratings corresponding to the present data were likewise not influenced by the optimism induction which could be a further indication that the effect of the experimental optimism induction had not yet resulted in any significant changes in pain experience as well as the accompanying catastrophizing thoughts (Basten-Günther et al., 2021). Moreover, it is thinkable that although no overall effect was found, certain subgroups may well have benefitted from the optimism induction, which will be discussed below.

While state measures of optimism and pain catastrophizing in our study were not associated, trait measures of optimism (*LOT-R*) and pain catastrophizing (*PCS*) were moderately (in the experimental group strongly and significantly) negatively correlated with each other. This confirms the above-mentioned findings by Hood et al. (2012) and Goodin et al. (2013) on the negative association of optimism with pain catastrophizing and underlines the need for differential analyses—and eventually treatments—taking into account prior individual differences or “risk factors” which render certain subgroups especially responsive for certain types of interventions.

### Influence of Optimism Induction on Situational Pain Catastrophizing Dependent on Level of Dispositional Pain Catastrophizing (Hyp.2)

Since the expected main effect of the optimism induction on situational pain catastrophizing was not found, it seems particularly interesting to look for moderating variables to identify possible subgroups which might nevertheless benefit from the optimism induction. To begin with, our model including dispositional pain catastrophizing (PCS) revealed that higher PCS scores were associated with higher situational catastrophizing (SCQ). This is in accordance with traditional state–trait theories and with prior empirical evidence (Quartana et al., 2009; Campbell, 2010a,b; Sturgeon and Zautra, 2013).

More importantly, in line with our hypotheses, there was a significant time x condition x PCS second order interaction effect, providing evidence that depending on participants’ level of trait pain catastrophizing, the optimism induction acts to different degrees on their situational pain catastrophizing. In other words, the association of PCS with SCQ scores varied dependent on experimental condition (optimism or control group) and time of measurement (pre vs. post-optimism induction). In order to get an impression on which difference(s) exactly this global second order interaction effect might be based on, correlations were calculated separately for both groups at both assessment times (pre/post). As shown by the correlation analyses, it descriptively appears that in the control group, PCS and SCQ scores are strongly and significantly correlated at both times of measurement. In contrast, in the optimism group, there is a similarly significant correlation in the first measurement but this correlation is lower and no longer significant after the optimism induction.

Regarding scores of situational pain catastrophizing, participants with low levels of dispositional pain catastrophizing tended to manifest comparably lower levels of situational pain catastrophizing, whether they received an optimism induction or not. In high trait pain catastrophizers, on the contrary, those in the BPS group would on average have a lower pre–post increase of situational pain catastrophizing than those in the TD group. This suggests that the impact of dispositional catastrophizing on situational catastrophizing is attenuated by the optimism induction. The trait’s manifestation in the actual pain situation (Tett and Guterman, 2000) seems to be counteracted by situational optimism. Thus, high dispositional pain catastrophizers benefitted more from the optimism induction as regards their situational pain catastrophizing than low dispositional pain catastrophizers. These results—though still wanting an inferential statistical testing in larger samples—provide preliminary results for resilience models according to which protective factors such as optimism buffer against risk factors such as pain catastrophizing (for example, Catalano et al., 2011).

Participants with *low* trait pain catastrophizing in the BPS condition descriptively showed a pre-post SCQ increase, while in the TD group, low trait pain catastrophizers’ situational pain catastrophizing remained almost the same. Possibly, the descriptively observed increase was caused by some participants who tended to downplay their catastrophizing thoughts in the PCS and the first SCQ measurement but were more open about these thoughts in the more optimistic state induced by the writing task. Thus, there would not have been a change in actual catastrophizing but only in the willingness to report it. A similar speculation was made concerning the pre–post increase in facial expression of pain in the BPS group in the study by Basten-Günther et al. (2021), which was interpreted as reflecting participants’ increased openness and readiness to communicate their pain after being made more optimistic.

Both optimism and pain catastrophizing have been ascribed a communicative, interpersonal function (Dougall et al., 2001; Brissette et al., 2008). As proposed by the “communal coping model,” pain catastrophizing could serve as a coping strategy aiming at amplifying pain experience and pain behavior in order to pursue relational goals such as maximizing proximity or soliciting assistance and empathic responses from one’s social environment (Keefe et al., 2000; Sullivan et al., 2001, 2004). It is thinkable that in optimists, pain catastrophizing is more dispensable as a coping strategy because optimism in itself leads to higher perceived social support (Dougall et al., 2001; Brissette et al., 2008) and possibly more trust in the social environment, which might subsequently provoke increased pain communication. Indeed, as reported above, the analysis of facial responses in the first part of the study (Basten-Günther et al., 2021) showed stronger facial expression of pain after the optimism induction. As most of the mentioned studies do not distinguish between state and trait optimism/catastrophizing, more research is needed to clarify to which of these concepts and in what way these considerations apply.

### Influence of Optimism Induction on Situational Pain Catastrophizing Dependent on Level of Dispositional Optimism (Hyp.3)

Including the LOT-R score as a covariate in the ANCOVA, there was no main or interaction effect of trait optimism regarding situational pain catastrophizing. Therefore, in accordance with prior findings (Harrist et al., 2007; Peters et al., 2010; Hanssen et al., 2013), trait optimists do not seem to have benefitted more or less from the optimism induction and trait optimism does not seem to have acted directly on situational pain catastrophizing in our study. Nevertheless, there was a negative correlation between trait optimism (*LOT-R*) and SCQ which was moderately strong and significant in the SCQ pre measurement but only weak and no longer significant in the post measurement. Regarding the two experimental conditions, it descriptively appears that only in the BPS group, the association falls from pre: *r* = −0.412 (*p* = 0.071) to post: *r* = −0.159 (*p* = 0.504; TD group: pre: *r* = −0.224, *p* = 0.343; post: *r* = −0.161, *p* = 0.497). These correlations might cautiously be interpreted as a hint for a compensatory mechanism: due to our optimism manipulation, the association between trait optimism and situational pain catastrophizing seems to be weakened which could mean that the optimism induction somehow levels out effects of prior interindividual differences in optimism. Consequently, participants low in dispositional optimism might benefit slightly more from the optimism induction compared with participants who dispose of high optimism regardless of the writing task, as a result of their general disposition to view things—including pain—in a more positive way. However, given the fact that these correlations were not significant and our hypothesis was not confirmed by the ANCOVA, these are only cautious speculations which would need to be explored in further studies.

### Limitations

The present study is to our knowledge the first to examine the effect of an experimental optimism induction on situational pain catastrophizing. While well-established measures for optimism and pain catastrophizing were used, it would still be interesting to compare our results with studies using different questionnaires such as, for example, the Coping Strategies Questionnaire (*CSQ*; Rosentiel and Keefe, 1983) to measure trait catastrophizing. It also has to be stressed that the BPS optimism induction does not aim at fostering unrealistic optimism, which is also called wishful thinking and could have adverse effects in pain conditions (Jefferson et al., 2017), but instead at increasing realistic optimism, leading to the maintenance of a positive outlook on life despite pain (Nes and Segerstrom, 2006; Esteve et al., 2007). Our sample was balanced with regard to sex and age and the experimental manipulation of optimism allows for causal conclusions and the control of many confounding variables. Referencing pain catastrophizing to a preceding experimental pain—compared to recalling a past clinical pain experience—provides a high standardization. It nevertheless has to be taken into account that the study only included healthy participants in an experimental setting. Therefore, it would firstly be interesting to examine the generalizability of our results to naturalistic, everyday contexts where pain experiences can be less predictable and less controllable than during experimental pain and therefore possibly provide more opportunity for catastrophizing thoughts and feelings or else to experimentally manipulate predictability and controllability. Secondly, applying the paradigm in clinical, possibly post-operative or chronic pain populations with higher levels of pain catastrophizing appears as a logical next step. While we did not find any association between catastrophizing and pain outcomes, this relation has been found in clinical populations which tend to display higher levels of pain catastrophizing (for example, Granot and Ferber, 2005). Furthermore, the relation between situational catastrophizing and *chronic* pain should be studied as well. It has to be stressed, however, that regardless of the effects on pain, catastrophizing in itself constitutes a large burden implying huge mental distress, which is way diminishing it must be seen as clinically relevant *per se* (Petrini and Arendt-Nielsen, 2020). However, it has to be kept in mind that on the individual level, not all participants responded to the optimism induction. A small number of participants showed no increase or even decreases in situational optimism. An investigation of these non-responders could provide useful insights. Lastly, it has to be acknowledged that our sample size was 40, which appears adequate given the high time expense and staff effort of conducting a 2-h experimental pain session, but nevertheless could constitute a certain risk for a lack of power in detecting effects, particularly with the second-order interactions. For this reason, results should be treated with some caution, and we strongly recommend validating our findings and testing the contrasts in the second-order interaction in studies with larger sample sizes.

### Conclusion

In the present study, the effect of an experimental optimism manipulation on situational pain catastrophizing was moderated by participants’ level of trait catastrophizing. Descriptively, it appeared that participants with higher trait pain catastrophizing benefitted more from the optimism manipulation in that they showed a less pronounced increase in situational pain catastrophizing than the control group. These results support resilience models stressing the buffering role of optimism. Further research is needed to validate the findings in larger sample sizes and to test the buffering function of optimism in everyday pain and in clinical populations. By developing preventive or therapeutic interventions which focus on subgroups at risk, optimism-fostering visualization techniques, which have been shown to lead to longer-term benefits when applied repeatedly (Peters et al., 2017; Molinari et al., 2018 for preliminary evidence in fibromyalgia patients; Malouff and Schutte, 2017; Carrillo et al., 2019 for meta-analyses), could become an important complement to existing cognitive-behavioral and other approaches.

## Data Availability Statement

The raw data supporting the conclusions of this article will be made available by the authors, without undue reservation.

## Ethics Statement

The studies involving human participants were reviewed and approved by the Ethikkommission, Otto-Friedrich-Universität, Bamberg. The patients/participants provided their written informed consent to participate in this study.

## Author Contributions

All authors listed have made a substantial, direct, and intellectual contribution to the work and approved it for publication.

## Conflict of Interest

The authors declare that the research was conducted in the absence of any commercial or financial relationships that could be construed as a potential conflict of interest.

## Publisher’s Note

All claims expressed in this article are solely those of the authors and do not necessarily represent those of their affiliated organizations, or those of the publisher, the editors and the reviewers. Any product that may be evaluated in this article, or claim that may be made by its manufacturer, is not guaranteed or endorsed by the publisher.

## Abbreviations

BPS: Best Possible Self
TD: Typical day
LOT-R: Life Orientation Test-Revised Version
PCS: Pain Catastrophizing Scale
SCQ: Situational Catastrophizing Questionnaire
FEX: Future Expectancies Scale
PANAS: Positive and Negative Affect Schedule
ANCOVA: Analysis of covariance
