# Supplementary material for: The Effect of Induced Optimism on Situational Pain Catastrophizing
Source: Front Psychol. 2022 Jun 23;13:900290. doi: 10.3389/fpsyg.2022.900290 (PMC9260170; doi:10.3389/fpsyg.2022.900290)

Supplementary material A: Regressions of situational pain catastrophizing (SCQ) on dispositional pain catastrophizing (PCS) for each condition (BPS vs. TD) at each time of measurement (pre vs. post).

*SCQ = Situational Catastrophizing Questionnaire. PCS = Pain Catastrophizing Scale. BPS = Best Possible Self (experimental group). TD = Typical Day (control group).*


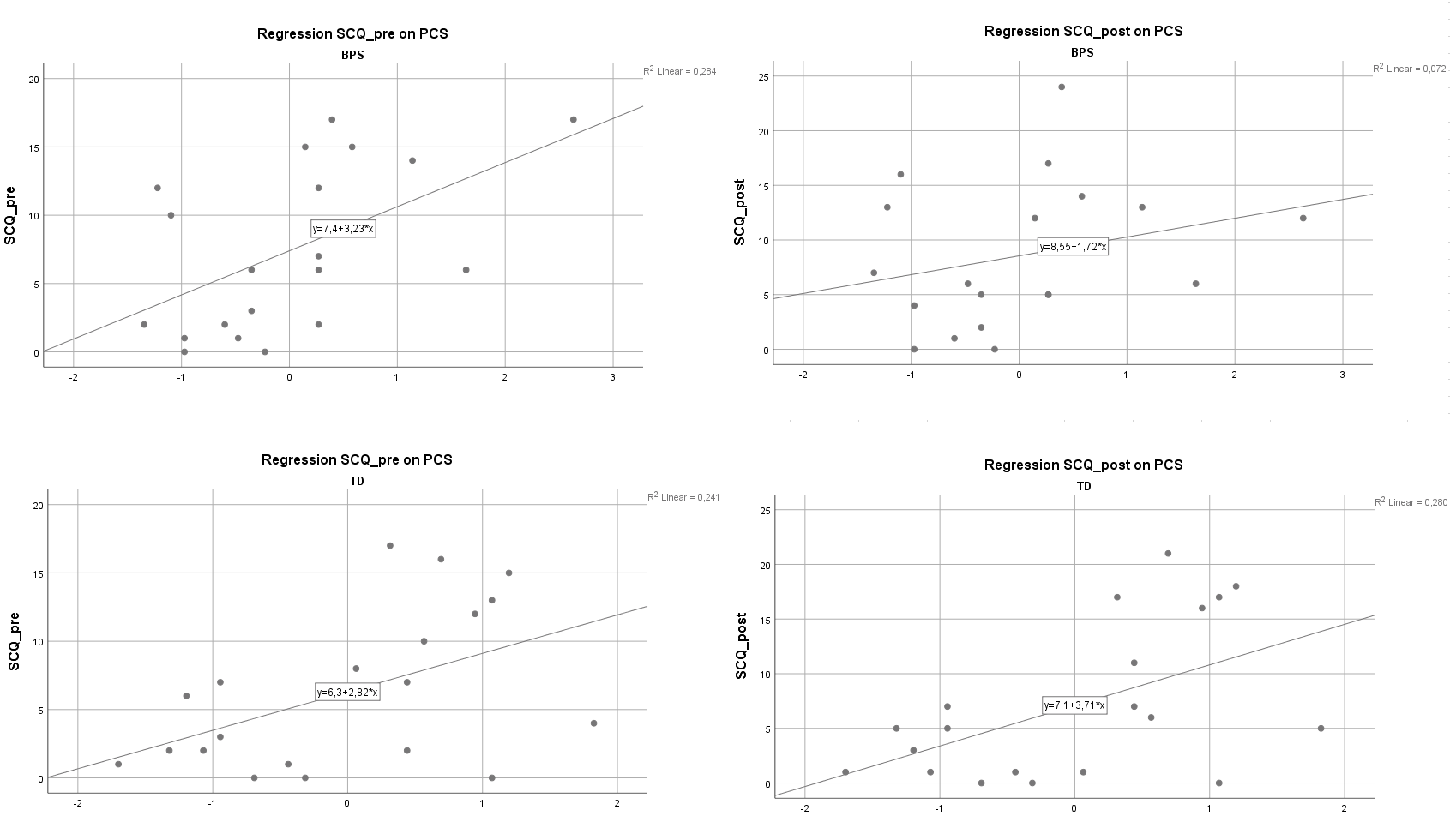

Supplement: Supplementary file 1 [file Table_1.DOCX]
